# Coping with Hypertension among Indigenous Peoples in Brazil and the Role of the Primary Care Nurse: A Critical Review from a Transcultural Perspective

**DOI:** 10.3390/nursrep11040086

**Published:** 2021-11-16

**Authors:** Mauricio Viana Gomes Oliveira, Ângela Maria Mendes Abreu, James R. Welch, Carlos E. A. Coimbra

**Affiliations:** 1 Departamento de Enfermagem, Universidade Federal de Rondônia, Porto Velho 76801-059, Brazil; 2 Escola de Enfermagem Anna Nery, Universidade Federal de Rio de Janeiro, Rio de Janeiro 20211-130, Brazil; angelamendesabreu@gmail.com; 3 Escola Nacional de Saúde Pública, Fundação Oswaldo Cruz (FIOCRUZ), Rio de Janeiro 21041-210, Brazil; welch@ensp.fiocruz.br (J.R.W.); carloscoimbrajr@gmail.com (C.E.A.C.J.)

**Keywords:** primary care nursing, indigenous people’s health, transcultural healthcare, hypertension, Brazil

## Abstract

Our objective is to critically review the literature addressing the strategic role of nurses in the daily primary care of arterial hypertension in Indigenous communities in Brazil. We selected studies based on an initial keyword search of major bibliographic indexing databases for the years 2000 to 2020 and manual search. Further selection was based on topical, methodological, and thematic relevance, as well as evaluation of scholarship quality and pertinence to our chosen narrative. The literature demonstrates Indigenous peoples do not receive health services that measure up to national standards in large part due to a marked lack of academic and employer preparation for nurses operating in transcultural settings. Inequities were apparent in recurrent reports of victim-blaming, deficient clinical communication with patients, clinical malpractice, devaluation of hypertension as a problem for Indigenous peoples, insufficient intercultural training for nurses, and discrimination against Indigenous students in nursing education programs. This systemic problem needs to be addressed by universities and the Indigenous Health Care Subsystem in Brazil.

## 1. Introduction

In Brazil, arterial hypertension is a major risk factor affecting the adult population. According to the 2008 National Household Sample Survey, nearly 14% of Brazilians aged 18 years or over self-reported having been diagnosed with hypertension [1]. In 2013, the prevalence of hypertension in the same population was 32.3%, with a sharp increase among adults 30 years and older [2].

Although Brazil is one of the countries with the largest Indigenous ethnic diversity in the world, its self-declared Indigenous population was just 0.4% of the national population in 2010 [3]. In the same year, a solid majority (57.7%) of Brazil’s Indigenous population lived in more than 600 federally recognized Indigenous lands, 98% of which by area was in the Amazon region (the remainder resided outside federal lands in both rural and urban settings). Health services to Indigenous residents of federal Indigenous lands are provided by the Indigenous Health Care Subsystem, under the umbrella of the Brazilian Unified Health System (SUS) [4]. The Subsystem delivers primary care at health posts usually located near one or more communities, often at great distances from the nearest hospital. The primary care nurse plays a strategic role in the functioning of the system, being responsible for conducting prenatal consultations, dispensing prescribed medications, monitoring chronic diseases, among many other important tasks. Very often the nurse is the only health professional with higher education at a health post, usually assisted by at least one bilingual Indigenous health agent, who is also essential for the workflow, especially when residents do not speak Portuguese [5,6].

The Indigenous population in Brazil has been undergoing a swift epidemiological transition, with chronic non-communicable diseases and violence becoming more pronounced causes of morbidity and mortality since the 1980s [7]. Additionally, territorial circumscription and encroachment by agribusiness compromise the physical and psychological well-being of Indigenous peoples. Food insecurity causes serious health problems, especially chronic undernutrition, and anemia during childhood. At the same time, economic, environmental, and political changes in Indigenous communities facilitate the consumption of industrialized foods rich in sugar, salt, and fat [8]. A consequence of these transformations is the rapid accumulation of elevated burden of chronic noncommunicable diseases, especially arterial hypertension, diabetes mellitus, and excess weight [7]. Although blood pressure assessment among Indigenous peoples in Brazil has been carried out sporadically in a few communities since the 1950s [9,10], it was only in 2008–2009 that it was investigated nationally. The First National Survey of Indigenous People’s Health and Nutrition in Brazil (henceforth ‘National Survey’) was the first study to evaluate blood pressure levels in a nationwide representative sample of Indigenous women (unfortunately, men were not included in the study) [7].

The National Survey reported an overall hypertension prevalence of 13.2% for nonpregnant adult women, being higher in the central-west (17.5%) and south/southeast (17.4%) regions of the country [7]. This is surprising because researchers who investigated blood pressure levels in the 1950s–1960s in the central-west region (Kalapalo, Kamaiurá, Karajá, Mundurukú, Xavante) found no cases of hypertension [9,10,11].

The objective of this article is to critically review recently published literature addressing the strategic role of nurses in the daily primary care of arterial hypertension in Indigenous communities in Brazil, emphasizing inequities involving transcultural dynamics. We address challenges deriving from the Brazilian Indigenous Health Care Subsystem primary care model and its difficulties attending to the enormous diversity of cultural and intercultural meanings of “hypertension” among the country’s many Indigenous ethnic groups. We also evaluate the adequacy for Indigenous peoples in Brazil of public policies regarding diagnosis, treatment, and prevention of hypertension; limitations and deficiencies of hypertension nursing as practiced in primary healthcare settings in Indigenous communities; and to what extent the Subsystem adequately prepares nurses to work with hypertension patients in transcultural settings.

## 2. Study Setting and Methods

**Figure 1:**
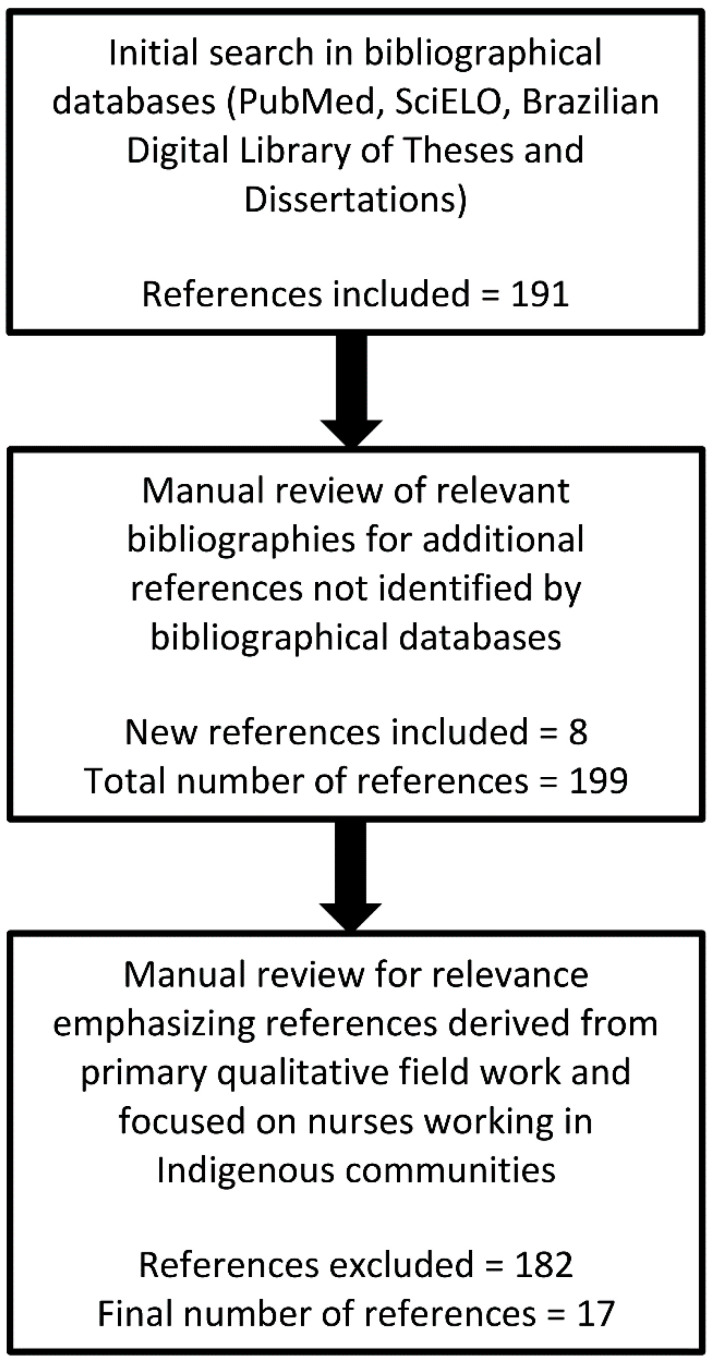
Flow chart of reference inclusion and exclusion per search and refinement methodology.

We conducted a critical review [12] of qualitative studies addressing nursing in primary care health posts attending to Indigenous communities situated in federally recognized Indigenous lands. We chose two bibliographic databases, considering the scope of their coverage of major Latin American journals in nursing and public health: PubMed and SciELO (Scientific Electronic Library Online). In addition, we consulted the Brazilian Digital Library of Theses and Dissertations. Databases were searched using the following MeSH (Medical Subject Headings) terms: nursing; public health nursing; education, Indigenous health services; Indigenous populations; Brazil. The PubMed search string was: ((nursing OR public health nursing) AND education AND (Indigenous health services OR Indigenous populations) AND Brazil). The SciELO and Brazilian Digital Library of Theses and Dissertations search strings were broadened due to the small number of results: (enfermagem) AND (indígena). The search included the period 2000 to 2020 to coincide with the initial consolidation of the Indigenous Health Care Subsystem [4]. The initial search was not meant to be exhaustive, as bibliographies of retrieved references were also searched for additional references that were not identified by our search parameters (Figure 1).

Retrieved references were reviewed for relevance, with an emphasis being given to papers that derived from primary qualitative fieldwork and focused on nurses working in Indigenous communities. Studies based on quantitative methods, bachelor’s dissertations, and review articles were excluded. Based on authors’ reading of this body of literature, four recurrent themes were identified as most relevant for the quality of nursing services accessed by Indigenous peoples: (1) primary care settings in Indigenous communities, (2) narrative accounts of nursing practice among Indigenous peoples, (3) Indigenous perceptions of hypertension, and (4) intercultural education and training of primary health care nurses working with Indigenous peoples. The selected literature was further refined based on the presence of content addressing these four themes. Consistent with critical review methods, the final selection of bibliographic references was determined by the authors according to their thematic value for the chosen narrative. Reference selection for discussion in this review was subjectively determined based on authors’ extensive knowledge of hypertension nursing in Indigenous communities in Brazil, their reading and interpretations of the body of literature meeting selection criteria, and their evaluations of the empirical quality of the references.

After identifying four main themes we wished to address in this critical review, and identifying literature with content addressing these themes, we reread the final literature selection in order to identify relevant first-hand qualitative data, pertinent interpretations and analyses, and illustrative quotations that would be most useful for communicating our argument. It should be noted that the final bibliographical selection exhibited substantial convergence in argumentation and messaging. These were then presented, sometimes quoted, discussed, and evaluated in our article according to the four themes identified earlier in the literature selection process. Thus, the arguments we present are our own, developed through intimate contact with the full range of literature available that addressing our topic. Where appropriate due to scarcity of published sources, we drew on our own field experiences to complement the discussion. The authors have trained health professionals, particularly nurses, in Indigenous peoples’ health from an interdisciplinary perspective for decades.

## 3. The Primary Care Setting in Indigenous Communities

The Indigenous Health Care Subsystem attending to the Indigenous population is organized into 34 local healthcare units, called Special Indigenous Health Districts. Each district delivers primary care by means of multidisciplinary health teams that, theoretically, should make regular visits to communities. According to Pontes and Santos [4], “[this] health-care model is based on the notion of ‘differentiated health care’, which advocates that health initiatives should consider the linguistic, sociocultural and geographical specificities of Indigenous territories. Furthermore, health actions should be undertaken in dialogue with Indigenous peoples to ensure Indigenous knowledge, practices and specialists are embedded in health programs and policies (p. i108)”.

However, in the daily experience of Indigenous communities, the model is often distant from reality. For example, it is usual for multidisciplinary teams to lack doctors and dentists. Also, transport vehicles are frequently inadequately maintained and lacking fuel for long trips. As a result, nurses are often isolated in communities, causing them to provide basic health care only with the assistance of Indigenous health agents [6].

## 4. Hypertension in Transcultural Perspective

From a biomedical viewpoint, hypertension is a chronic, symptom-free disease, diagnosed when repeated blood pressure readings exceed the predefined cut-off points of 140 mmHg for systolic pressure and/or 90 for diastolic pressure. Given its ‘silent’ condition, a considerable number of people are unaware of having hypertension, which puts them at higher risk for cardiovascular diseases.

Anthropological studies carried out among non-Indigenous populations in different regions of Brazil point to hypertension etiologies that do not always converge with those of biomedicine. According to Uchôa [13], “… the disease experience cannot be considered as a simple reflection of the pathological process in the biomedical sense of the term; it should be conceived as a cultural construction that is expressed in specific manners of thinking and acting” (p. 102). In this sense, case studies provide examples of multiple and non-exclusive cultural models explaining hypertension in terms of such factors as “stress,” worry, fear, anger, poor diet, and nervous breakdowns [14,15]. Notwithstanding the predominant biomedical view that hypertension is usually asymptomatic, folk knowledge in diverse populations “fills in the gaps” with common sense symptoms. Thus, cultural models of hypertension experiences may identify such symptoms as headache, dizziness, tiredness, and body pain, even though these are not recognized by biomedicine as common symptoms that define hypertension. These cultural models may influence the degree to which patients adhere to treatments prescribed by the biomedical health professionals.

Similarly, we have found primary care nurses attending to Indigenous peoples who encounter a lack of adhesion to treatment protocols may attribute it to lack of (biomedical) understanding or cultural “interference” (Indigenous treatments that do not comport with biomedical therapies). However, the reality is much more complex and involves the totality of the intercultural medical exchange, including how health professionals understand and communicate with local Indigenous people. Specifically, nurses may assume that dialog about hypertension causes and treatments with members of another culture who often speak native languages is impossible or impractical considering demands on their work time. However, negligence of this kind prevents patients from fully understanding the biomedical rationale of diagnosis and treatment and prevents them from making fully informed decisions. This failure is especially problematic for asymptomatic diseases, such as hypertension, that are diagnosed according to opaque measurements by potentially unfamiliar instruments (sphygmomanometers) with abstract cut-off points. These chronic diseases also present special challenges for intercultural communication because disease in many cultures is understood in terms of experiences of unwellness (pain, weakness, fever, etc.), and treatments are considered successful and diseases cured when the perception of wellness returns [16,17,18].

In successful intercultural health settings, especially at the primary care level, disease and health knowledge is co-constructed through ongoing dialog. Nurses must seek knowledge of their patient’s cultural viewpoints and be prepared to accommodate them, even if they conflict with their own understandings. For example, an anthropological study of diabetes among the Pima in the southwestern United States showed the problem of high disease prevalence to be highly complex, involving political, economic, and cultural factors [19]. The study concluded that effective prevention and treatment programs required cultural sensitivity and community participation in addition to structural changes to healthcare services. Furthermore, these programs benefit from the exchange of cultural knowledge, such that health professionals and Pima co-constructed knowledge about the disease and its local circumstances.

A similar message emerged from a study of hypertension among black women in New Orleans [20]. According to the author, the cognitive worlds of health professionals and patients influence how they understand illness and affect one another through intercultural exchange. Former areas of disparity are thereby transformed into new understandings and areas of congruence. Thus, conventional unilateral educational interventions cannot be expected to increase treatment adhesion among Indigenous peoples. Rather, they exacerbate chronic miscommunication with Indigenous patients and thereby stimulate poor treatment adhesion. According to Weaver [21], “… If a client is delivered a message in a way he/she cannot fully take in, the true message is lost. Don’t give your people lip service, serve your people well” (p. 201).

## 5. Case Studies of Nursing in Practice among Indigenous Peoples in Brazil

By analyzing a selection of ethnographic case studies carried out among Indigenous peoples throughout Brazil, it is possible to identify some common points that help clarify the ideological gap separating nurses from the Indigenous populations they serve.

A study undertaken among the Mura in Amazonia revealed an absence of dialog between nurses and patients [22]. Upon the arrival of an Indigenous patient to the health post, the nurse did not thoroughly listen to the individual or conduct a physical examination. Rather, they rapidly gave some medication (e.g., syrup, pain killer) before promptly ending the consultation. According to the author, “… delivery of these medicines was always accompanied by the number of times this Indigenous person should take the medicine, but with little explanation …” [22] (p. 99). It is not surprising that, in the continuation of the interview between the researcher and the same nurse, she claimed to know “that the [Mura] would not take the prescribed medicines and that if they visited the Indigenous people’s homes, they would find the same medicines [unused], as the Indians do not follow prescriptions” [22] (p. 99).

Another study conducted among the Munduruku [6], also in Amazonia, describes a similar situation. A recently graduated nurse who received from her employer no information about the Indigenous community to which she was assigned assumed a distant and procedural posture in her interactions with patients, only receiving them at the health post and avoiding visiting Indigenous households. This professional’s consultations were restricted to measuring temperature and arterial blood pressure before quickly giving the Indigenous health agent a small note with the medicine to be delivered. According to the author, “… the dialogue established by the nurse presupposed total adherence to the forms of care offered. Non-adherence was understood to be a lack of self-care” by the Indigenous person [6] (p. 201).

The third study differs from the first two in addressing the case of a young and idealistic nurse who wished to care for people from other cultures. She moved from São Paulo to the Upper Xingu, a multiethnic Indigenous land in Amazonia [23]. Unlike the nurses described in the previous studies, this nurse prepared for her move by taking a course addressing basic anthropology and epidemiology. According to the author, the greatest challenge faced by this nurse “… consisted of learning to deal with her own values as an ‘Occidental person, Catholic, and health professional’ … [reconciling] the ‘history of … lifesaving at all costs’ with Indigenous values regarding life” [23] (pp. 518–519). Immediately upon first visiting the Kalapalo community, this nurse was faced with a great challenge that marked her deeply—a newborn child already considered dead by family members and community leaders but who still showed vital signs (heartbeat and weak breathing). For this nurse, this child’s life needed to be saved at any cost, including traveling to the nearest health post, with better resources, located many hours away by boat. After insistent conversations with family members, leaders, and the shaman, the nurse managed to secure permission to take the child to the health post. Midway along the trip, the shaman turned to the nurse and asked: “So, my daughter, is it dead yet?” The nurse then replied: “no, but she will die, she will die. You were right” (p. 521). The child died in her lap, on the boat, long before arriving at the post [23].

The selected studies highlight limitations of intercultural training and information about the culture and modus operandi of Indigenous societies provided to nurses contracted by the Indigenous Health Care Subsystem. In the studies presented above, the culture, customs, and beliefs of Indigenous peoples are interpreted as being among the greatest hurdles to providing more adequate care to Indigenous patients. Very similar reports are encountered in other articles based on qualitative research conducted with other ethnic groups [6,24,25,26,27]. The gap that separates nursing professionals from their served communities generates perceptions about Indigenous people as “lacking hygiene,” not always understanding Portuguese, not following prescriptions, missing scheduled appointments, complaining, and repeatedly returning with the same problem.

Interestingly, anthropological studies in Brazil show a quite different perspective of Indigenous people in relation to biomedicine and its professionals. For example, among the Wari’ in the state of Rondônia, an in-depth ethnographic investigation shows that this Indigenous group does not consider their own traditional medical system and biomedicine to be antagonistic. According to Conklin [28], “… the fundamental disagreements with Occidental medicine do not necessarily reside in the efficacy of treatments, but in the organizational structure of the health services. Wari’ society is highly egalitarian and basic health knowledge is socialized … In contrast, the occidental medical science present among the Wari’ is based in specialized practices and resources, hierarchically organized, and administered by outsiders” (p. 183), thus distancing itself from the community. Among the Baniwa of the Upper Rio Negro, Garnelo and Wright [29] demonstrate how “… myths that explain the origin of illnesses show the existence of diverse traditional categories of disease that orient traditional healing practices and the incorporation of biomedical knowledge. Baniwa cosmology operates like a reception system for biomedicine information, which is appropriated and resignified according to the logic of mythical thought” (p. 281). Other studies undertaken among different ethnic groups in Amazonia show that in general Indigenous peoples are receptive to medicines provided by biomedical professionals, although they may come to be reinterpreted according to the Indigenous peoples’ own concepts regarding curative substances [30,31,32].

## 6. Indigenous Perceptions of Hypertension

Research measuring blood pressure levels among Indigenous populations in Brazil from the 1950s to the 2000s (the earliest of which were cited in Introduction) was strongly influenced by the thesis that relative isolation from “Western civilization” was a protective factor for arterial hypertension [33]. The consolidation of this biomedical vision presents practical implications extending to the present, since many professionals continue to think that hypertension is not a priority for health planning and interventions among Indigenous communities.

Certain characteristics of hypertension, including its symptomatic invisibility, opaque diagnosis criteria, and relatively recent emergence among Indigenous peoples in Brazil make it practically impossible for most people to identify it without visiting health services. For the same reasons, it is no simple task to study Indigenous perceptions of the disease, which has yet to fully engage with the collective imagination through ongoing discourse with health professionals [29,34]. Although anthropological studies specifically addressing hypertension among Indigenous peoples in Brazil are scarce, we present below several emblematic examples.

A study conducted among the Kaingang in southern Brazil reveals important aspects related to Indigenous perspectives of hypertension selfcare, treatment, and curing [35]. The local biomedical protocol for prescribing antihypertensives is an important factor that generates conflict between the health team and individuals presenting hypertension. According to Kaingang perspectives, continued use of “pharmacy medications” weakens the body, making the person more susceptible to contracting diseases [36]. During her research, the author observed the existence of three nonexclusive forms of hypertension selfcare and treatment: medications from the health post, “forest remedies” and teas, and “prayers.” According to the author, the Kaingang cultural viewpoint that hypertension can be cured helps explain an interviewee report: “I was hypertensive and, since I don’t like to take medications, I controlled it with tea … I began to feel better … and perceived that I no longer felt this dizziness or headache, I cured myself and also stopped taking the tea.” Religious influence in the curing process is marked in the Kaingang context, as illustrates the testimony of another interviewee: “The Holy Spirit sent me the cure, because the Lord is all-powerful, and if it is his will, we are cured … . I never again had problems with high pressure” [36] (pp. 108–109).

Pharmaceuticals tend to be well accepted in Indigenous societies, whether or not other therapeutic avenues are also followed. According to Langdon [37], “our medicine is generally received positively, [the greater challenge falls to] the professionals in offering differentiated care, respecting Indigenous cultures” (p. 123). Research shows that Indigenous people seek out biomedicine as one among various therapeutic alternatives such as herbalists, shamans, and spiritists [38,39,40]. This pattern may frustrate a nurse who provides primary care to Indigenous communities and, due to lack of intercultural training, devalues the agency and decision-making capacity of Indigenous patients. The unfortunate but documented eventuality of health professionals speaking directly against traditional Indigenous belief systems and medical practices generates distrust. This distrust compounds the problems of authoritative dispensing of medicines, inadequate adequate explanation and dialog, and long-term prescriptions for asymptomatic chronic diseases. Like the Kaingang example described above, the Xukuru in northeast Brazil and the Parecí and Xerente in Central Brazil are similarly reported to place particular emphasis on traditional family selfcare practices [41,42,43].

Medical pluralism is the coexistence of distinct medical traditions with their own cultural logics, such as traditional and biomedical systems, in the medical itineraries of members of a society [44,45]. In recognition of and respect for medical pluralism among Indigenous peoples, the Brazilian National Indigenous Health Care Policy advocates for “differentiated attention,” which includes valuing traditional medical systems and, to the degree possible, their intersection with biomedicine [4,46]. In practice, however, research suggests nurses working with Indigenous communities strongly resist respecting the complex interrelationships that exist between Indigenous and biomedical traditions in the daily practices of patients and their families. According to Langdon and Garnelo [47], “… the common sense of many health professionals who work with Indigenous groups understands ‘culture,’ ‘beliefs,’ and ‘tradition’ as static realities and in this way interpret them as obstacles for the acceptance of biomedical services” (p. 465).

A lack of intercultural sensitivity preparation of nurses who work with Indigenous populations can perpetuate what is already a major structural separation between Indigenous and biomedical viewpoints. This cultural distance is a built-in challenge for primary health services that can negatively impact even routine preventive and treatment actions. The intercultural challenge is even greater when a disease such as hypertension is diagnostically opaque and requires lifelong treatment including potentially disagreeable lifestyle changes, such as reduction in salt consumption, quitting smoking or drinking alcoholic beverages, and adoption of physical activity routines. In our evaluation, these factors conspire against treatment compliance by patients who do not receive adequate explanation through respectful dialog and thereby may feel that they and their cultural values lack appropriate consideration by primary care nurses responsible for assisting their communities.

## 7. Intercultural Education and Training of Primary Health Care Nurses Working with Indigenous Peoples

In their daily work, nurses live with a great paradox. On the one hand, they are meant to be guided by the fundamental tenet of Indigenous healthcare policy, which “… advocates that health initiatives should consider the linguistic, sociocultural and geographical specificities of Indigenous territories” [4] (p. i108). On the other hand, they are underprepared by their universities and employers to put their nursing knowledge into practice in unfamiliar cultural contexts. Recent studies of primary care nurses working with Indigenous communities in Brazil have shown that the most recurrent points were communication difficulties due to language differences and lack of understanding of Indigenous cultures leading to biases against traditional selfcare practices [6,48,49,50]. According to Reis and Borges [48], the nurse’s “lack of preparation for intercultural work [causes] the biomedical treatment model to predominate” in community health posts, with ‘culture’ being viewed as an obstacle to their work (p. 181).

In view of the deficiencies typical of nurse education and preparation, some nursing programs in Brazil have sought to offer undergraduate curriculum options that prepare students for work in cross-cultural contexts. These efforts are incipient when compared to educational institutions in other countries, such as the USA, Canada, and Australia, where intercultural training for nurses working with Indigenous communities has been consolidated at least since the mid-twentieth century [21,51,52]. In these countries, Indigenous nursing students are also more common than in Brazil.

An example of an innovative intercultural pedagogical experience in Brazil is a three-month nursing internship available at the Federal University of Amazonas, during which students join a primary health care team working in an Indigenous community [53]. Another strategy for training nurses in intercultural practice has been observed in some universities offering a typically optional course in basic anthropology or social science. Nevertheless, analysis of intercultural training in the curricula of 69 nursing programs in public and private universities in the Amazon region shows that “… the majority of these institutions address the topic in diluted form, by means of an evasive concept of interdisciplinarity …” [54] (p. 6).

Recently, Indigenous people of different ethnic affiliations have completed nursing programs, many benefitting from affirmative action policies benefitting Indigenous and other minorities in the public universities. According to a study undertaken in Central Brazil [55], nursing programs are among the most sought after by Indigenous candidates because these youth “… want to do the best for their communities and, in a very expressive way, perceive that ‘nurses from outside’ remain very little time [in community health posts] due to the lack of adaptation to the locale …” (p. 78). The training of Indigenous nurses could contribute to overcoming the cultural “barriers” that frequently limit effective intercultural nursing performance. However, it is not enough for a student to be Indigenous; training programs require structuring according to the educational agenda of transcultural health service [55,56,57]. It is important to emphasize that even Indigenous nurses working in community health posts participate in intercultural or transcultural contexts, as they navigate the dynamic frontiers between Indigenous and biomedical health practices, beliefs, and practices.

Studies conducted among students and professors of nursing programs mainly located in Amazonia are unanimous in concluding that nursing programs are not well prepared to receive Indigenous students. This is because professors are not prepared for teaching nursing from a transcultural perspective and schools lack pedagogical approaches that adequately contemplate students’ diverse historical, sociocultural, environmental, and political backgrounds. Thus, hegemonic biomedical practices predominate while traditional medical systems and ethnomedicine are relegated to a secondary level [55,56,57].

The dilemma described above is well illustrated by the case of an Indigenous nursing student at a public university in the state of Mato Grosso who, upon being asked about the preparedness of the institution and its professors to attend to Indigenous students, responded “I don’t see preparation here. First, because the knowledge about indigenous peoples of [professors] who work here is very poor … many times an Indigenous student will have some difficulty in communicating, and the professor is not able to understand the students’ manner of expression, and this compromises their academic performance. I also perceive that our student colleagues in class often think it is strange and question why Indians are studying nursing” [55] (p. 91). A similar comment was documented by Quadros [58], who interviewed a Terena nursing student in the state of Mato Grosso do Sul: “[my student colleagues at college] were unfriendly towards us [Indigenous students] … there were barriers … so some girls did not talk with us” (p. 52). These examples also show that prejudice regarding Indigenous people in Brazil is a complex process, demonstrated here by ill-prepared professors and students who shun Indigenous students in class. Unfortunately, existing affirmative action policies are insufficient to overcome historical injustices suffered by Indigenous peoples in Brazil.

## 8. Conclusions

Recent reviews of the health profiles of Indigenous peoples worldwide provide evidence of a profound gap separating Indigenous societies from their corresponding non-Indigenous national populations [59]. In the Brazilian case, this inequity is particularly pronounced, considering major recent advances in principal health indicators attained by the general population and indications that the Indigenous population has not benefited equally [7,60].

Working as a primary care nurse in Indigenous communities in Brazil presents enormous challenges since, as seen in the case studies presented in previous sections, the great majority of these professionals are not sufficiently trained to put transcultural nursing into practice in Indigenous contexts. In addition, based on our field experience and this literature review, it is evident that the Indigenous Health Care Subsystem lacks a protocol for nurses who accompany Indigenous hypertensive patients.

Different from infectious and parasitic diseases that are highly endemic in the country’s Indigenous lands and usually have treatment protocols with clearly defined start and finish dates (e.g., malaria, tuberculosis), hypertension control has no predetermined end. As we discussed in the bibliographic review, many nurses who work at Indigenous health posts do not indulge in detailed explanations of the disease for Indigenous patients, limiting their interaction to distributing medications. Based on the articles reviewed here, we conclude that the overall practice of nurses who work in the Indigenous Health Care Subsystem fails to respond to the real health needs of the population served and is not consistent with the Brazilian National Indigenous Health Care Policy that advocates for differentiated attention.

Differentiated health care requires that primary health care for Indigenous peoples should function in consonance with the community, thus ensuring that Indigenous knowledge and practices are respected and culturally appropriate care is provided. Unfortunately, this basic governing principle is rarely achieved in practice. This is a systemic failure that involves nurses insofar as they are insufficiently trained to administer healthcare in transcultural settings and may not be allocated adequate work time to provide the personalized attention these contexts require, including proper explanations of biomedical perspectives of a disease like hypertension.

The last section of this review may be the most impactful because, based on the cited articles, it is apparent that nursing students in Brazil receive very little preparation for transcultural settings. In such an ethnically diverse country as Brazil, it is challenging for nurses to establish a two-way dialog between biomedicine and local knowledge traditions. Although the entrance of Indigenous students to public university nursing programs has provided hope that this problem will be addressed, unfortunately, reports by Indigenous students show universities are ill prepared to receive them. Indigenous nursing students are relegated to the institutional margins where they, alone, face difficulty communicating with professors and encounter racial prejudice from their peers. Such challenges and adversities faced by Indigenous university students may be associated with the rarified presence of Indigenous nurses in employment. According to a study characterizing the sociodemographic profile of nurses in Brazil, of a total of 414,712 professionals registered with the Brazilian Federal Council of Nursing, just 0.3% were identified as Indigenous, with the greater part being white (57.9%) or black (37.9%) [61].

This critical review addresses nurses’ roles in providing transcultural care in locations where they lack the support of complete multi-professional health teams. We focus on hypertension, a relatively recent illness to afflict the Indigenous population in Brazil. Additionally, being asymptomatic and uncurable, hypertension presents challenges for Indigenous populations with their own medical systems to buy into. Articles, book chapters, theses, and dissertations are organized according to four themes: the primary care setting in Indigenous communities, hypertension in transcultural perspective, case studies of nursing in practice among Indigenous peoples in Brazil, and Indigenous perceptions of hypertension. The cited literature demonstrates that Indigenous peoples do not receive health services that measure up to national standards in large part due to a marked lack of university and employer preparation for nurses operating in transcultural settings. Patients suffer as a result, even though the blame is often placed on them for low adhesion to prescribed biomedical treatments. Hypertension care among culturally distinct populations is a special challenge due to the disease’s invisibility and incurability, which may lead some patients to rely on their own cultural understandings of health and disease. Nevertheless, medical pluralism is common among Brazil’s Indigenous peoples, affording properly trained primary care nurses the opportunity to practice biomedicine while working alongside Indigenous medical systems. The systemic problem of lack of appropriate training in transcultural nursing needs to be addressed by universities and the Indigenous Health Care Subsystem in Brazil.
